# Body composition changes in resistance-trained adults Following 8-Weeks of supplementation with a combination of plant protein and creatine monohydrate

**DOI:** 10.1080/15502783.2025.2550213

**Published:** 2025-08-25

**Authors:** Joesi M. Krieger, Kevin F. Holley, Alex C. Schrautemeier, James L. Tice, Joshua Iannotti, Anthony M. Hagele, Connor J. Gaige, Wyatt B. McLaughlin, Ralf Jäger, Chad M. Kerksick

**Affiliations:** a Lindenwood University, Exercise and Performance Nutrition Laboratory, St. Charles, MO, USA; b Increnovo, LLC, Whitefish Bay, WI, USA

**Keywords:** Protein source, body composition, lean mass, protein, creatine

## Abstract

**Introduction:**

Adequate dietary protein supports body composition changes while resistance training. Due to its complete amino acid profile, whey protein has routinely outperformed plant-based options. However, when dosed effectively, plant-based protein can offer comparable benefits. Creatine monohydrate is well established for its ability to heighten resistance training adaptations. This study assessed changes in body composition over 8-weeks in resistance-trained males and females consuming a plant protein blend containing creatine monohydrate.

**Methods:**

A double-blind, placebo-controlled, randomized trial was conducted with 60 resistance trained males (*n* = 31; 26 years, 81.5 ± 8.8 kg, 179.2 ± 7.8 cm) and females (*n* = 29; 23 years, 66.3 ± 9.4 kg, 161.0 ± 12.4 cm). Participants were randomly assigned (*n* = 15 each group) to supplement with either 48 g of plant protein blend + creatine (PPCr), 48 g of plant protein blend (PP), 49 g of whey protein (WP), or 48 g of carbohydrate placebo (CHO) for 8 weeks, while following a structured 4-day per week resistance training program that emphasized strength and muscle hypertrophy. Body mass (BM) and body composition parameters, fat free mass (FFM), fat mass (FM), and percentage body fat were assessed using DEXA and total body water (TBW), intracellular water (ICW), and extracellular water (ECW) were assessed using bioelectrical impedance spectroscopy (BIS) at Week 0 and Week 8. A 4 × 3 mixed factorial ANOVA with repeated measures was used to evaluate differences. A p-value of 0.05 was used to establish statistical significance and trends were considered if p-values were between 0.05 and 0.10.

**Results:**

After 8 weeks of supplementation, no significant group × time interactions (*p* > 0.05) were observed for any of the measured variables. A trend was observed (*p* = 0.067) for intracellular water. Follow-up pairwise comparisons highlighted statistically greater increases (*p* = 0.004) for ICW in the PPCr (0.86 ± 0.64 L, 3.2%, d = 0.17) when compared to PLA (0.27 ± 0.47 L, 0.99%, d = 0.05). As evidence of a sound response to the exercise training program, significant main effects of time (*p* < 0.05) were observed for BM, FFM, TBW, ICW, and ECW (*p* < 0.001). There were no significant differences between groups for any other body composition variables.

**Conclusion:**

All groups experienced favorable body composition changes over time, with no major differences between protein sources. PPCr may influence ICW shifts, warranting further investigation using a longer intervention and larger sample sizes.

